# Automated Detection of Stereotypical Motor Movements in Autism Spectrum Disorder Using Recurrence Quantification Analysis

**DOI:** 10.3389/fninf.2017.00009

**Published:** 2017-02-16

**Authors:** Ulf Großekathöfer, Nikolay V. Manyakov, Vojkan Mihajlović, Gahan Pandina, Andrew Skalkin, Seth Ness, Abigail Bangerter, Matthew S. Goodwin

**Affiliations:** ^1^Holst Centre, IMECEindhoven, Netherlands; ^2^Janssen Research & DevelopmentBeerse, Belgium; ^3^Janssen Research & DevelopmentTitusville, NJ, USA; ^4^Janssen Research & DevelopmentSpring House, PA, USA; ^5^Department of Health Sciences, Northeastern UniversityBoston, MA, USA

**Keywords:** autism, ASD, recurrence plots, repetitive behavior, stereotypical motor movement, recurrence analysis

## Abstract

A number of recent studies using accelerometer features as input to machine learning classifiers show promising results for automatically detecting stereotypical motor movements (SMM) in individuals with Autism Spectrum Disorder (ASD). However, replicating these results across different types of accelerometers and their position on the body still remains a challenge. We introduce a new set of features in this domain based on recurrence plot and quantification analyses that are orientation invariant and able to capture non-linear dynamics of SMM. Applying these features to an existing published data set containing acceleration data, we achieve up to 9% average increase in accuracy compared to current state-of-the-art published results. Furthermore, we provide evidence that a single torso sensor can automatically detect multiple types of SMM in ASD, and that our approach allows recognition of SMM with high accuracy in individuals when using a person-independent classifier.

## 1. Introduction

Autism Spectrum Disorder (ASD) is a complex neurodevelopmental disorder characterized by common behavioral and social characteristics that can significantly impact daily living for individuals with ASD and their families. About 1 in 68 children by age eight are currently being diagnosed with ASD, wherein it is 5 times more common among boys (1 in 54) than girls (1 in 252) (Baio, 2012). Lifetime costs for one individual with ASD is estimated to be more than 2.3 million (Ganz, 2007), compared to 0.36 million spent on a child without the disorder (Alemayehu and Warner, 2004). Reducing the burden of ASD on both families and society is limited as a result of the great heterogeneity in symptom presentation seen across the autism spectrum, and reliance on behavioral observation rather than objective biomarkers for diagnosing the condition and evaluating intervention outcomes. More objective and efficient measures are needed in order to stratify subtypes within the ASD population, develop more targeted and effective therapies and drugs, and evaluate their success remediating core symptoms.

The Diagnostic and Statistical Manual of Mental Disorders, Fifth Edition (DSM-5, 2013) identifies two main criteria for diagnosing ASD: (a) persistent deficits in social communication and social interaction across contexts, and (b) restricted, repetitive patterns of behavior, interests, and/or activities. One of the ways restricted and repetitive behaviors manifest in ASD is stereotypical motor movements (SMM) that appear to observers to be invariant in form, having no obvious eliciting stimuli, and no adaptive function (Baumeister and Forehand, 1973). The most frequent forms of SMM observed in ASD are hand flapping, body rocking, and finger flicking. According to a recent review (Goodwin et al., 2011), between 60 and 100% of individuals with ASD exhibit at least one form of SMM.

Reliably and efficiently detecting and monitoring SMM over time could provide important insights for understanding and intervening upon a core symptom of ASD. SMM can negatively affect a child with ASD's development. If it becomes a dominant behavior in a child's repertoire, which it often does in ASD, it can interfere with learning and acquisition of new skills. Timely detection, characterization, and appropriate interventions can reduce the impact SMM have on learning and development. However, efficient and accurate monitoring of SMM is needed to reliably and validly assess which therapies and/or drugs are efficacious across the autism spectrum and over time. Continuous and objective monitoring capabilities are also needed to elucidate the mechanisms that drive SMM, which may include physiological, affective, and environmental factors.

Traditional measures of SMM primarily include general rating scales, either as part of a diagnostic tool, such as the Autism Diagnostic Observation Schedule-Second Edition (Lord et al., 2012) or Autism Diagnostic Interview- Revised (ADI-R, Le Couteur et al., 2003), or as part of a specific measure like the Repetitive Behavior Scale Revised Lam and Aman (RBS-R, 2007). While useful for documenting presence or absence of SMM, these measures rely on clinician interviews, limited behavioral observation, and/or parental report, all of which can be subjective, inaccurate, and difficult to compare across different individuals with ASD. Extended direct behavioral observation may provide a more objective alternative to these methods; however, it too can be inaccurate given limited samples of time to make observations and the difficulty documenting precise timing of SMM as it happens (Sprague and Newell, 1996; Gardenier et al., 2004; Goodwin et al., 2011). Video-based observation methods are more accurate at behavioral classification than interviews, rating scales, and direct observation, but they are time consuming in that they require detailed off-line annotation of videos. In addition, both direct and video measures of SMM are usually based on observations in controlled environments (i.e., lab or clinic), not in natural environments (i.e., home or classrooms). Taken together, there is a need for less obtrusive and more objective methods that allow continuous monitoring of SMM over time in naturalistic settings.

A number of investigators have begun to use wearable accelerometers and pattern recognition classifiers to develop better and more efficient measures of SMM in ASD. Extracting a standard set of movement features from one or more 3-axis accelerometers worn on fingers, wrists, and/or the torso while individuals with ASD engage in SMM, and feeding those features to commonly used machine learning techniques (e.g., Support Vector Machines; SVM), have yielded promising results for automatically detecting different types of SMM. Standard feature sets found to perform well in this domain to-date typically include: lower and higher order statistics, spectral components, correlation, entropy, and signal peak zero crossing number. An important caveat to this prior work is that while automated detection accuracy has been relatively high in specific ASD populations, generalizing these results using different accelerometer types and positions, has proved to be more challenging (Goodwin et al., 2014).

In the current work, we introduce novel features to this domain, namely, recurrence plots (Eckmann et al., 1987). Recurrence plots have been used in autism research for quantification of social interaction (Fusaroli et al., 2014), social motor coordination (Romero et al., 2016), as well as EEG-based diagnosis of autism (Bhat et al., 2014). The main advantage of recurrence plots over features traditionally explored are that: (a) they capture non-linear aspects of SMM, and (b) they are unaffected by orientation of acceleration sensors. As a result, and evidenced in the results reported herein, they can provide a robust way of detecting SMM that is not dependent on a particular type of accelerometer or where it is positioned on the body while recording. As reported in more detail below, we employed random forest to complementary SVM and decision tree methods, and to evaluate the usefulness of recurrence plots in the automated SMM detection domain. We found that this method is both highly accurate and useful in evaluating the contribution of each feature uniquely to the detection task.

Our proposed method was applied to an open-access data set previously collected and published by Goodwin et al. (2014). The purpose of the current study was three-fold. First, we sought to determine whether it is possible to improve on state-of-the-art published recognition accuracy using novel features and machine learning techniques in this domain. Second, we sought to identify the most useful features to enable optimization of accelerometer sensor type and placement for future recordings. Third, we aimed to estimate the reliability and robustness of features captured by accelerometer sensors positioned at different body parts.

In the following, we provide an overview of current research in automated recognition of SMM in ASD. We then describe in detail recurrence plots and recurrence quantification analyses we used for feature extraction. Next, we briefly review the data used for our analyses, followed by a detailed report of experiments we performed on the data. To evaluate our results, we focus on accuracy, training size, and sensor position. Finally, we present and discuss our results, ending with conclusions, and opportunities for future work.

## 2. Related work

Existing approaches to automated monitoring of SMM are based either on webcams or accelerometers. In a series of publications (Gonçalves et al., 2012a,b,c) a group from the University of Minho created methods based on the Kinect webcam sensor from Microsoft. Although, their approach shows promising results, the authors restricted themselves to detecting only one type of SMM, namely hand flapping. In addition, the Kinect sensor is limited to monitoring within a confined space and requires users to be in close proximity to the sensor. This limits the application of the approach, as it does not allow continuous recording across a range of contexts and activities.

**Figure 1:**
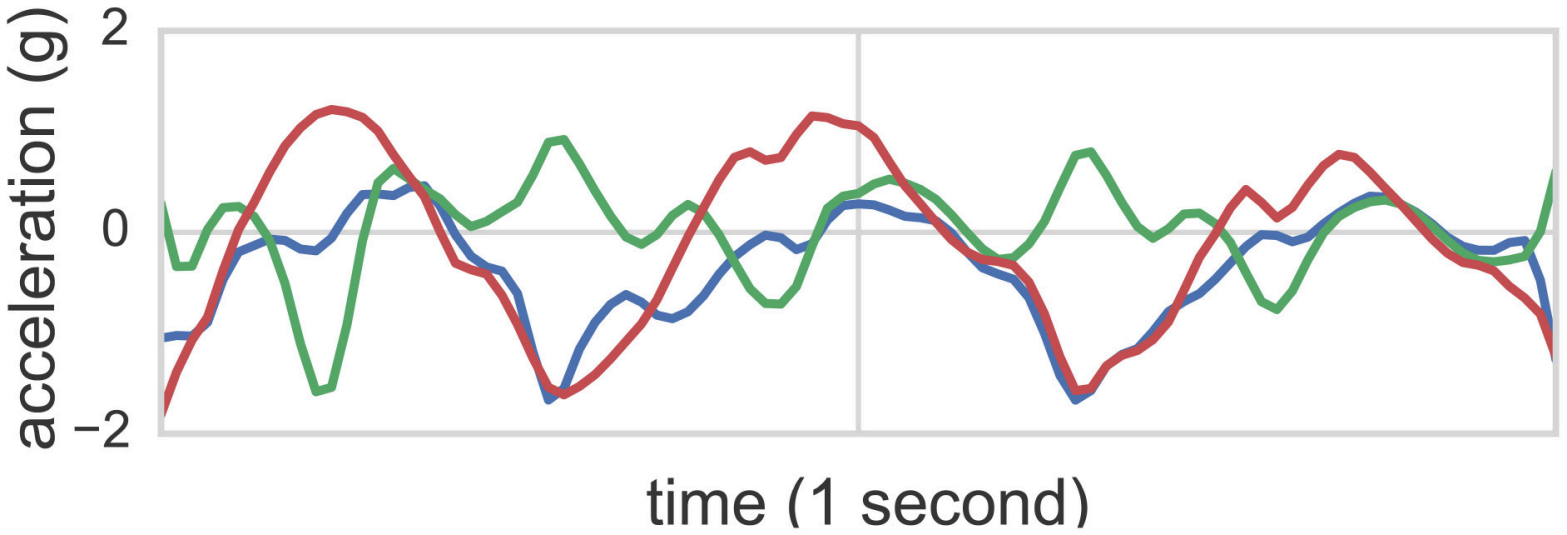
**Accelerometer readings of one second in length from the class *flapping***. The accelerometer was mounted to the right wrist. Each line corresponds to one of the three acceleration axes.

Alternative approaches to the Kinect are based on the use of wearable 3-axis accelerometers (see Figure 1). Although, the primary aim of previously published accelerometer-based studies is to detect SMM in individuals with ASD, some studies have been carried out with healthy volunteers mimicking SMM (Westeyn et al., 2005; Plötz et al., 2012)^1^, and therefore do not necessarily generalize to the ASD population.

To-date, there have been two different approaches to automatically detecting SMM in ASD using accelerometer data. One approach is to use a single accelerometer to detect one type of SMM, such as hand flapping when a sensor is worn on the wrist (Gonçalves et al., 2012a; Rodrigues et al., 2013). The second approach is to use multiple accelerometers to detect multiple SMM, such as hand flapping from sensors worn on the wrist, and body rocking with a sensor worn on the torso (Min et al., 2009; Min and Tewfik, 2010a,b, 2011; Min, 2014). Other studies have done the same, but included a detection class where hand flapping and body rocking occur simultaneously in time (i.e., “flap-rock,” see Albinali et al., 2009, 2012; Goodwin et al., 2011, 2014).

While more sensors appear to improve recognition accuracy in these studies, one practical drawback is that many individuals with ASD have sensory sensitivities that might make them less able or willing to tolerate wearing multiple devices. To accommodate for different sensory profiles in the ASD population, it would be ideal to limit the number of sensors to a minimum, while still optimizing accurate multiple class SMM detection.

Typical features used for acceleration analyses of SMM in prior studies have focused on: distances between mean values along accelerometer axes, variance along axes directions, correlation coefficients, entropy, Fast Fourier Transform (FFT) peaks, and frequencies (Albinali et al., 2009, 2012; Goodwin et al., 2011, 2014), Stockwell transform (Goodwin et al., 2014), mean standard deviation, root mean square, number of peaks, and zero crossing values (Gonçalves et al., 2012a; Rodrigues et al., 2013), and skewness and kurtosis (Min, 2014; Min and Tewfik, 2011). These features are mainly aimed at characterizing oscillatory features of SMM as statistical characteristics of values distributed around mean values in each accelerometer axis, joint relation of changes in different axial directions, or frequency components of oscillatory moves. While useful in many regards, these features fail to capture potentially important dynamics of SMM that can change over time, namely, when they do not follow a consistent oscillatory pattern or when patterns differ in frequency, duration, speed, and amplitude (Goodwin et al., 2014). A final limitation to previous publications in this domain, is that different sensor types have been used across studies. These may have different orientations, resulting in features with different values, despite representing the same SMM. To overcome this limitation, other sets of features are required that do not vary in their characteristics across different types of SMM and sensor orientations.

## 3. Methodology

### 3.1. Recurrence plots features

Rather than considering changes in accelerometer recordings from oscillations along each accelerometer axis separately, we propose to characterize these changes from the recurrence point-of-view and locally consider similarity between trajectories in phase space. In other words, we represent an accelerometer recording ${\vec{a}}_{t}=\left(x_{t},y_{t},z_{t}\right)$ as a trajectory in 3D space and analyze where it recurs to nearly the same position. With such an approach, similar SMM events will produce similar phase space trajectories, regardless of oscillation patterns or offsets. By considering a sliding window in time with length *T*, we are able to characterize SMM via trajectory recurrences in phase space for a short time interval (which covers only one SMM incident). Recurrence metrics allow us to locally detect SMM even if the pattern changes from event to event. As we are aiming for local characterization, changes in amplitudes between SMM events should not result in much disturbance to our recurrence assessment. Additionally, as shown further below, this approach is not dependent on accelerometer axis orientations and their changes because one can simultaneously consider all three axes of an accelerometer in phase space.

**Figure 2:**
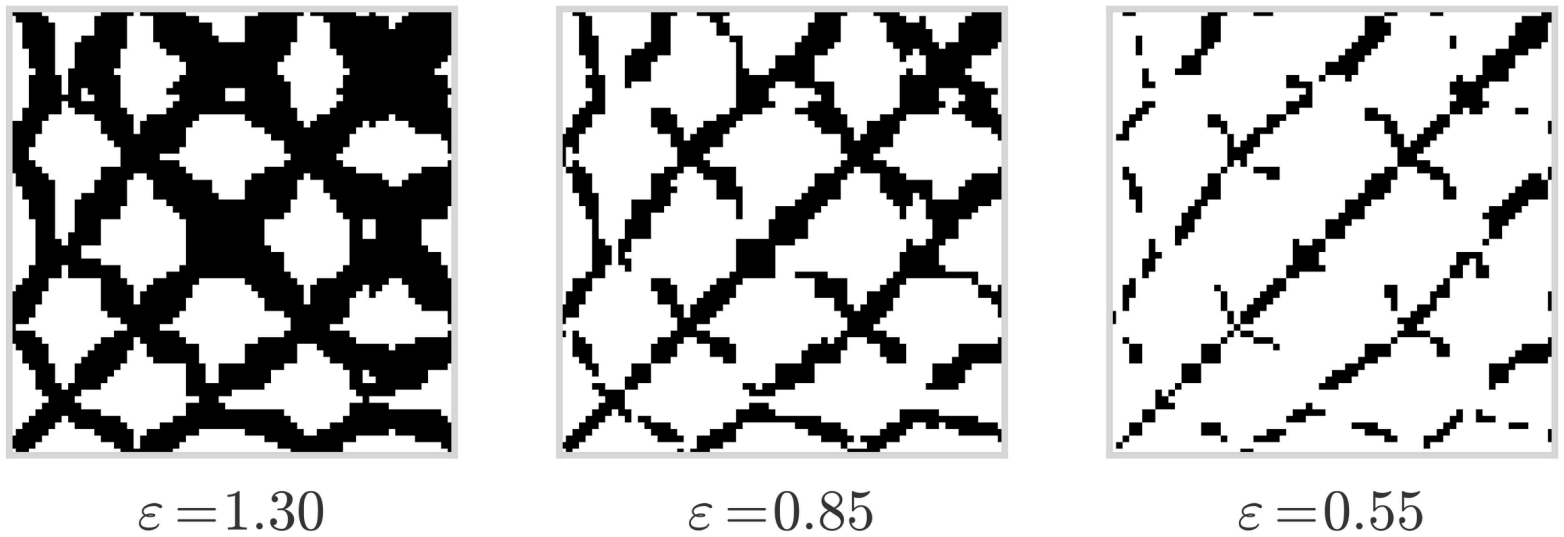
**Recurrence plots computed from the data displayed in Figure 1 and for three different values of the threshold parameter ε**.

Recurrence plots (Eckmann et al., 1987) are represented as quadratic matrices whose elements describe if a phase space trajectory returns to a location it has visited before. The elements are associated with two points in time in which the trajectory was at the same place in a phase space, or gets sufficiently close to a point it has been previously. As a result of the dynamics of the trajectory, a recurrence plot contains small-scale structures such as single dots, diagonal lines, and vertical lines that, in combination, result in large-scale structures that characterize an underlying system. As illustrated in Figure 2, a visual representation of recurrence matrices as plots, where all recurrence points are denoted as black dots, can reveal insights about the chaotic vs. deterministic nature of the phase space trajectory in question.

Recurrence plots are computed by comparing distances between all points of a trajectory

(1) $$R(i,j)=\Theta (\varepsilon -||{\vec{a}}_{i}-{\vec{a}}_{j}||).$$

Here, *R* is the matrix of recurrence points, Θ is the Heaviside step function, and ||·|| is a norm. For our analysis, we chose Euclidean distance.

Since a rotation *Q* is an isometry in Euclidean space, i.e.,

(2) $$||Q{\vec{a}}_{i}-Q{\vec{a}}_{j}||=||{\vec{a}}_{i}-{\vec{a}}_{j}||,$$

the distances in Equation 1 are identical. Thus, the recurrence matrix *R* is also invariant to rotations in the space.

However, measurements from accelerometers contain two components: acceleration relative to the containing space $\vec{a}$, as well as earth acceleration $\vec{g}$, i.e., gravity.

(3) $$\left||Q({\vec{a}}_{i}+\vec{g})-Q({\vec{a}}_{j}+\vec{g})||=||({\vec{a}}_{i}+\vec{g})-({\vec{a}}_{j}+\vec{g})||=||{\vec{a}}_{i}-{\vec{a}}_{j}|\right|$$

In this case, the distances are independent of the given rotation matrix *Q* and the influence of gravity $\vec{g}$, and the resulting recurrence matrices are invariant to orientation.

### 3.2. Recurrence quantification analysis

Recurrence plots provide a good visual characterization of a dynamic system, however, they cannot be directly used as features. It is desirable to further describe the number and duration of recurrences in order to allow the application of recurrence plot features in standard machine learning approaches. Such descriptors are known as *recurrence quantification analysis (RQA)* and multiple measures have been proposed (Webber and Zbilut, 1994; Marwan et al., 2002; Naschitz et al., 2004). They are based either on the density of recurrence points in a recurrence plot, or the number and frequency of diagonal and vertical lines. For further information on recurrence plots, please refer to the overview by Marwan et al. (2007).

In our experiments, we used the following RQA measures to quantify the recurrence plots from accelerometer readings:

- **Recurrence rate (RR)** describes the density of recurrence points in a recurrence plot, i.e., the fraction of recurrence points over the total amount of points.
- **Determinism (DET)** is the ratio of recurrence points which form diagonal lines in the recurrence plot. In contrast to chaotic processes, deterministic behavior yields less isolated recurrence points, and longer diagonals; the length of the lines is thus related to the inverse of the largest positive Liapunov exponent (Eckmann et al., 1987).
- **Laminarity (LAM)** represents the occurrence of laminar states in the system without describing the length of these laminar phases.
- **Ratio (RATIO)** can be used to uncover transitions in the dynamics: during certain types of qualitative transitions RR decreases, whereas DET remains constant (Webber and Zbilut, 1994).
- **Averaged diagonal line length (L)** represents the average time that two segments of a trajectory are close to each other.
- **Trapping time (TT)** estimates the mean length of vertical lines, similar to L. This value corresponds to the mean time a system will stay in a specific state and how long the state will be trapped.
- **Longest diagonal line (L**_max_**)** is related to the exponential divergence of the phase space trajectory: the faster the trajectory segments diverge, the shorter the diagonal lines.
- **Longest vertical line (V**_max_**)** are able to find chaos-to-chaos transitions (Marwan et al., 2002) and allow for the investigation of intermittency, even for rather short and non-stationary data (Marwan et al., 2007).
- **Entropy (ENTR)** reflects the complexity of the recurrence plot in respect to the diagonal lines, e.g., for uncorrelated noise the value of ENTR is rather small, indicating low complexity.

Table 1 summarizes these RQA features in detail.

**Table 1:** **Overview of the RQA features used in this study**.

**RQA measure name**	**Equation**
Recurrence rate	RR $=\frac{1}{N^{2}}\overset{N}{\underset{i,j=1}{\sum }}R\left(i,j\right)$
Determinism	DET $=\frac{\overset{N}{\underset{\ell =\ell_{\min}}{\sum }}\ell P\left(\ell \right)}{\overset{N}{\underset{i,j=1}{\sum }}R\left(i,j\right)}$
Laminarity	LAM $=\frac{\overset{N}{\underset{v=v_{\min}}{\sum }}vP\left(v\right)}{\overset{N}{\underset{v=1}{\sum }}vP\left(v\right)}$
Ratio	RATIO $=N^{2}\frac{\overset{N}{\underset{\ell =\ell_{\min}}{\sum }}\ell P\left(\ell \right)}{{\left(\overset{N}{\underset{l=1}{\sum }}P\left(\ell \right)\right)}^{2}}$
Average diag. length	L $=\frac{\overset{N}{\underset{\ell =\ell_{\min}}{\sum }}\ell P\left(\ell \right)}{\overset{N}{\underset{\ell =\ell_{\min}}{\sum }}P\left(\ell \right)}$
Trapping time	TT $=\frac{\overset{N}{\underset{v=v_{\min}}{\sum }}vP\left(v\right)}{\overset{N}{\underset{v=v_{\min}}{\sum }}P\left(v\right)}$
Entropy	ENTR $=-\overset{N}{\underset{\ell =\ell_{\min}}{\sum }}p\left(\ell \right)\ln\;p\left(\ell \right)$
Longest diag. line	${\text{L}}_{\max}=\max\left({\left\{\ell_{i}\right\}}_{i=1}^{N_{\ell }}\right)$
Longest vert. line	${\text{V}}_{\max}=\max\left({\left\{v_{i}\right\}}_{i=1}^{N_{v}}\right)$

*ℓ and v are the length of diagonal and vertical lines in recurrence plots, respectively, ℓ_min_ and v_min_ are the minimal diagonal and vertical length that should be considered. The total number of diagonal and vertical lines of lengths ℓ and v is expressed as histograms P(ℓ) and P(v), respectively. While p(ℓ) and p(v) describe the estimated length distributions, $N_{\ell }=\underset{\ell \geq \ell_{min}}{\sum }P\left(\ell \right)$ is the total number of diagonal lines and, similarly, N_v_ the total number of vertical lines*.

Note that the results of RQA are subject to the chosen threshold value ε (see Equation 1). On one hand, if ε is made too large, almost all points are in the ε-neighborhood of all other points, resulting in a recurrence plot which is too dense. On the other hand, if ε is made too small, one may not be able to identify any recurrence points and corresponding structures (Marwan, 2011), see Figure 2. In our analyes, we treat ε as a hyperparameter during model selection (cf. Section 5.1).

### 3.3. Decision tree classifier

Decision Trees (DTs) are a supervised learning method used for classification (Breiman et al., 1984). The goal is to create a model that predicts the value of a target variable by learning simple decision rules inferred from data features that can be represented as a search tree. While DTs are able to learn a model that is simple to understand and interpret, they are prone to overfitting that might result in poor generalization properties. Several algorithms exist to automatically extract DTs from a set of observations. We use the python implementation of the CART algorithm to learn a DT as provided by the scikits-learn python package (Pedregosa et al., 2011) with Gini splitting rule and constructed a tree until all leaves are pure.

### 3.4. Random forests classifier

Random forest classifiers (RF) are an ensemble method for classification that combine predictions of multiple DT classifiers (Breiman, 2001). RF classifiers are easy-to-use, yield state-of-the art performance (Fernández-Delgado et al., 2014), and scale to very large data sets.

The training algorithm for RF applies the general technique of bagging to tree learners. Given a training set, bagging repeatedly selects a bootstrap of *n* sample and fits DTs to these samples. In addition to the bagging procedure, RF uses a modified tree-learning algorithm that selects, at each candidate split in the learning process, a random subset of features. As it is typically done, we use $\sqrt{d}$ features in each split based on Gini splitting rule, where *d* is the total number of features. After training, predictions for unseen samples can be made by taking the majority vote of the DT ensemble.

Typical values for the number of trees range from a few hundred to several thousand, depending on the size and nature of the training set. Training and test error tend to reach a stable level after some number of trees have been fit. An optimal number of trees can be found using cross-validation, or by observing out-of-bag error: the mean prediction error on each training sample, using only trees not included in the bootstrap sample.

Based on the selection of a random subset of features, RF enables variables to be ranked in order of importance for classification problems, in a natural way. If one or a few features are very strong predictors for the target output, these features will be selected in many of the trees, causing them to become correlated.

To measure the importance of the *j*-th feature after training, the values of the *j*-th feature are permuted in the training data and out-of-bag error is again computed. The importance score for the *j*-th feature is computed by averaging the difference in out-of-bag error before and after permutation over all trees. The score is normalized by the standard deviation of these differences. Features which produce large values for this score are ranked as more important than features which produce small values.

### 3.5. Support vector machines

SVMs are binary classifiers to distinguish two classes (Burges, 1998; Vapnik, 1998). SVMs aim to minimize the empirical risk of an incorrect classification by means of a separating hyperplane. The idea of SVMs is to constrain the capacity of a learned hyperplane function in order to maximize its generalization properties. SVMs incorporate a hyperparameter *C* by which value miss-classifications are penalized during training. For a detailed introduction to SVMs refer to Burges (1998). In the experiments described below, each feature was normalized to z-score with respect to the training data.

## 4. Data set

In order to directly compare results from prior studies, we performed our analyses on data described and made publicly available in Goodwin et al. (2014). Wherever we compare results between the current paper and those reported in Goodwin et al. (2014), we followed the same exact process for generating training and testing sets.

Data were collected from 6 children between the ages of 12 and 20 years recruited from The Groden Center, RI–a school for children an adults with autism and other developmental difficulties (cf. Table 2 for participant characteristics). All participants were diagnosed with ASD and had a significant score on the RSB-R (Lam and Aman, 2007) for body rocking and/or hand flapping. The same individuals participated in two studies, three years apart, referred to here as Study 1 and Study 2. The study was approved by a human subjects review board and parental consent including permission to release deidentified data for additional data analyses by additional parties for research purposes was obtained for each participant.

**Table 2:** **Participant characteristics including total score of Childhood Autism Rating Scale (CARS)**.

**Participant**	**Age**	**Sex**	**Diagnosis**	**CARS**
1	14	Male	ASD	42
2	14	Male	ASD	33
3	13	Male	ASD	43.5
4	16	Male	ASD	39
5	20	Male	ASD	36
6	13	Male	ASD	38

During both studies, participants wore three 3-axis accelerometers, one in a band on each wrist, and the other on the torso, secured with a strip of fabric around the chest. Different accelerometers were used in each study. Study 1 used MITes 3-axis sensors recording ±2 g data at 60 Hz. Study 2 used Wockets 3-axis sensors recording ±4 g data at 90 Hz. Recordings were done across multiple sessions within each study.

While wearing sensors, participants were video recorded wherein video time was synchronized with sensor time. These recordings were consequently annotated offline by two behavioral science experts, where time segments noting hand flapping (*flap*), body rocking (*rock*), and simultaneous rocking and flapping (*flap-rock*) start and end times were labeled. These annotations (with 90% joint agreement between two independent raters achieved) served as ground truth labels for 3+1 (3 types of stereotyped motor behavior + non stereotyped activity) classification tasks. Figure 1 illustrates accelerometer readings of one second length from the class flapping. The accelerometer was mounted to the right wrist of participant 1. Each line corresponds to one of the three acceleration axes.

Table 3 lists descriptive statistics on our dataset, including total length of combined sessions in which data were collected from participants, number of different SMM observed, and total SMM durations. A *bout* describes a contiguous time range in which an individual engaged in SMM behavior.

**Table 3:** **Overall SMM statistics in our dataset calculated using manual video annotation**.

**Participant**	**Sessions**		**No. of bouts**			**Dur. bouts (min)**		
	**No**.	**Dur. (min)**	**Rock**	**Flap**	**Flaprock**	**Rock**	**Flap**	**Flaprock**
**STUDY 1**								
1	2	50.2	52	2	57	5.2	0.2	4.9
2	2	32.1	1	209	0	0.1	8.1	0
3	2	64.5	1	52	28	0.1	2.9	2.4
4	2	38.9	55	70	0	12.7	6.2	0
5	2	44.7	12	75	17	1.2	8.6	1.6
6	2	55.8	112	0	9	31.3	0	1.6
**STUDY 2**								
1	3	49.8	77	57	64	7.4	6.5	5
2	2	51.1	77	33	1	7.7	1	0.1
3	2	75.9	1	18	0	0.02	0.5	0
4	3	87.5	46	38	0	13.2	2	0
5	2	55.2	68	11	6	10.9	1.3	0.3
6	1	25.3	64	0	6	20.2	0	6

*The left three column donates the participant number, followed by the number of sessions the participant was observed and the combined duration of all sessions in minutes. Columns three to five summarize the number of bouts per SMM class the participant engaged in during sessions, while columns six, seven, and eight denote the combined duration of the SMM behavior in minutes*.

To reduce influence of class skewness resulting from different amounts and durations of the SMM classes in the data, we followed an identical balancing scheme as suggested in Goodwin et al. (2014). We used balanced data for training and natural imbalanced data for testing; balancing the data was done by randomly under-sampling the majority class (i.e., unknown) and re-sampling the minority classes (i.e., SMM).

We accessed the data set at http://cbslab.org/smm-dataset/.

## 5. Experiments

We designed an experimental setup to investigate the following research questions:

- **Accuracy:** are RQA features for accelerometers with different classifiers able to represent SMM with high accuracy?
- **Generalization:** can a classifier trained using all but one participant's data accurately classify SMM movements in the left-out participant?
- **Training size:** how many observations are needed to train a classifier before high accuracy is achieved on the participant left out?
- **Sensor position:** which sensor position has the most accuracy to detect these three classes of SMM?
- **Feature importance:** which RQA features contribute the most to describing differences between SMM and non-SMM movements?

### 5.1. Experiment 1: accuracy

In order to assess the accuracy of RQA features for detecting SMM, we reproduced the experimental procedure described in Goodwin et al. (2014). Here, data from all sessions within each study for each participant was combined. Subsequently, a *k*-fold cross-validation was performed such that *k* is the number of sessions a participant was observed within each study, and every fold consists of data from a specific session. For this experiment, we extracted RQA features for each available accelerometer stream (one for each wrist and one on the torso), resulting in *d* = 27 features. Feature extraction was performed identical to those described in Goodwin et al. (2014): data streams were segmented with *T* = 1s windows and an overlap between consecutive data segments of 87%. As classifiers, we used (i) RF classifiers for which we selected the number of trees *n*_*trees*_ ∈ {100, 250, 500} during cross-validation; (ii) linear SVMs for which we evaluated the optimal penalty weight *C* ∈ {1, 100, 10, 000, 100, 000} also during cross-validation; and (iii) DT classifiers. To select the optimal ε-values (Ohgi et al., 2007; Marwan, 2011) for RQA feature extraction, we treated ε as a hyperparameter in cross-validation with ε ∈ {2·0.65^*i*^|*i* = 0, …, 15}. In order to allow a comparison of our findings with previously published results, we used all four annotated behavior classes (i.e., *rock, flap, flap-rock, non-stereotyped*). All experiments were implemented in the Python programming language; the SVM implementation provided by libsvm (Chang and Lin, 2011) was used, which incorporates a one-vs.-one scheme to address multi-class classification.

### 5.2. Experiment 2: training size

The experimental setup followed a leave-one-participant-out validation scheme: all data from Study 1 was combined across all sessions and the classifiers were trained on data from all except one participant. The data from the remaining participant was then used for testing. This was done 6 times, once for each participant. To determine minimum required training size to achieve stable accuracy in a leave-one-out-participant validation scheme, we created additional training sets of decreasingly fewer training examples from the original training data available during cross-validation. The number of training examples is relative to the total amount of training data according to a *training set size factor K*. For example, if the total number of training examples is *N* = 1, 000, then the actual number of examples used in training for a training set size factor *K* = 0.5 is *N*_*K* = 0.5_ = 500. Note that *N*_*K*_ was rounded to the closest natural number if necessary. In total, we evaluated 15 different values for training set size factor *K* with *K* ∈ {0.5^*i*^|*i* = 0, 1, …, 14} for each training data set.

### 5.3. Experiment 3: sensor position and feature importance

In order to evaluate which sensor position contributed most to accurately detecting SMM, we designed an experimental setup where a combination of all three accelerometer streams and different sensor positions were evaluated separately. The data dimensionality for the latter was *d* = 9 in this experiment (i.e., x, y, and z for 3 sensors). Again, we followed the protocol from Experiment 1, but limited our analysis to data from Study 1.

Subsequently, we further investigated which features yielded the highest feature importance values in the trained RF classifiers comprising the optimal number of trees found in Experiment 1.

## 6. Results and discussion

### 6.1. Accuracy

Table 4 summarizes the results from Experiment 1. We first sought to estimate a possible increase in performance using the new feature set compared to the features used in prior analyses of the current data set Goodwin et al. (2014). For this reason we followed the same training procedure and incorporated the same classifiers as presented in Goodwin et al. (2014). Columns denoted with SVM_GW_, and DT_GW_ show highest results reported in Goodwin et al. (2014) for SVM and DT classifiers. The result with the new set of features are shown in columns indicated with RF_RQA_, SVM_RQA_, and DT_RQA_.

**Table 4:** **This table shows the classification accuracies from Experiment 1**.

**Participant**	**Classifier**				
	**RF_RQA_**	**SVM_RQA_**	**SVM_GW_**	**DT_RQA_**	**DT_GW_**
**STUDY 1**					
1	0.83	0.82	**0.87**	0.77	0.79
2	**0.89**	0.87	0.85	0.81	0.80
3	0.93	0.93	**0.94**	0.92	0.89
4	**0.91**	**0.91**	0.66	0.87	0.48
5	0.80	**0.81**	0.75	0.79	0.71
6	**0.88**	**0.88**	0.84	0.82	0.81
**STUDY 2**					
1	**0.80**	0.79	0.71	0.77	0.62
2	0.69	0.68	**0.80**	0.65	0.72
3	**0.99**	**0.99**	**0.99**	**0.99**	**0.99**
4	**0.95**	0.93	0.90	0.91	0.90
5	**0.85**	**0.85**	0.73	0.84	0.69
6	−	−	−	−	−

*Columns labeled with RF, SVM, or DT indicate results from random forest, support vector machines, or decision tree classifiers, respectively. Columns subscripted with RQA show results from our RQA features, while columns with subscripted GW show best results reported in Goodwin et al. (2014). Note that the latter represents the highest classification accuracy selected from 3 different feature sets for both classifiers. Since participant 6 engaged in only one session in Study 2, we cannot report leave-one-session-out cross-validation results.Bold values indicate best classification accuracy in each row (i.e., for each subject and for each study)*.

In 8 out of 11 analyzed cases, our approach yielded higher accuracy values as compared to classifiers that used standard features. For DT classifiers, our approach yielded on average ≈ 0.83 accuracy, while DT_GW_ showed on average ≈ 0.76 (an increase of ≈ 9.2%). For SVM classifiers, our RQA feature set reached an averaged classification accuracy of ≈ 0.86, while the features used in Goodwin et al. (2014) yielded ≈ 0.82 (equals an increase of ≈ 5%). In some cases, classification accuracies for both feature sets are almost identical (e.g., participant 3, Study 2), however, differences of more than 40% points can be observed (participant 4, Study 1). With an average accuracy of >0.86, RF classifiers yield a slightly higher classification accuracy than SVMs.

### 6.2. Generalization

**Figure 3:**
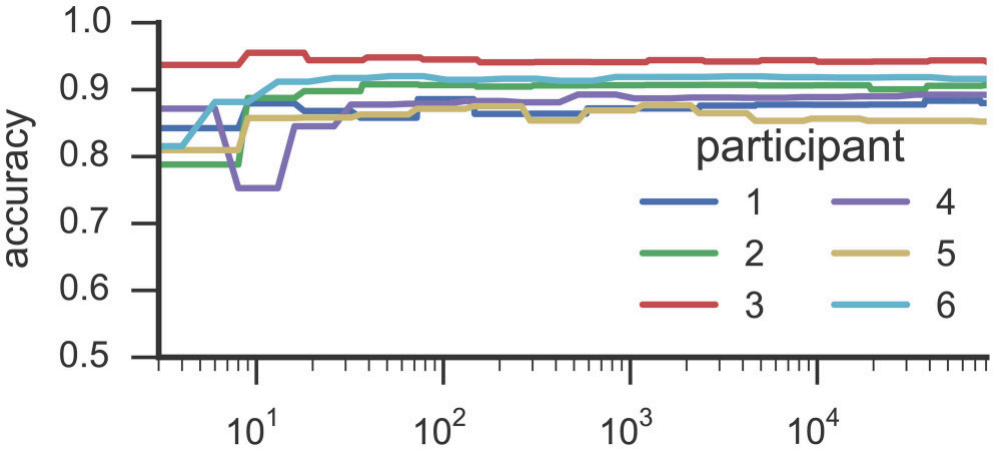
**This figure illustrates the results from Experiment 2**. Each line characterizes the dependency between the training set size (y-axis) and the reached accuracy (x-axis) for one participant by means of RF classifiers. The accuracy values were derived in a *k*-fold cross-validation where the folds correspond to the recording sessions of Study 1. The training examples where randomly selected from all available training data.

**Figure 4:**
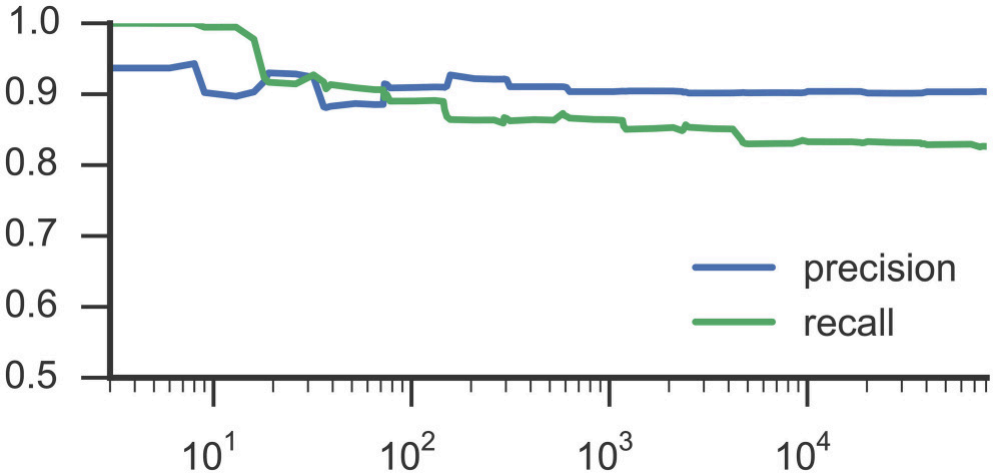
**This plot shows average precision and recall values based on training set size, corresponding to Figure 3 and RF classifiers**.

Figures 3, 4 list results from Experiment 2. In Figure 3, each line characterizes the dependency between the training set size (x-axis) and achieved accuracy (y-axis) for one participant, which was excluded from training. Figure 4 illustrates precision and recall values from the same experiment averaged across all 6 participants. We found that classifiers reached good classification accuracy for data from each left out participants, i.e., were able to generalize over participants. These findings suggest that it is possible to deploy a system that incorporate a pre-trained classifier that can be used to accurately recognize SMM without further requirements for personalization or adaption to a particular user.

### 6.3. Training size

Results from the experiment toward training size (see Figure 3) suggests that <100 training examples are sufficient to train a RF classifier that is able to identify SMM with a stable level of accuracy on data from participants who were unknown to the classifier in training. Adding more examples increased classification performance only marginally. Some participants' movements could be classified with substantially fewer training examples. For example, results for participant 3 reached a stable plateau of ≈ 0.87 classification accuracy with only 32 training examples. For participant 6, a classifier trained with only 19 examples yielded an accuracy value of ≈ 0.94. However, the performance of classifiers trained with such a low number of training examples heavily depends on which training examples were randomly selected while sub-sampling the data set.

### 6.4. Sensor position

**Figure 5:**
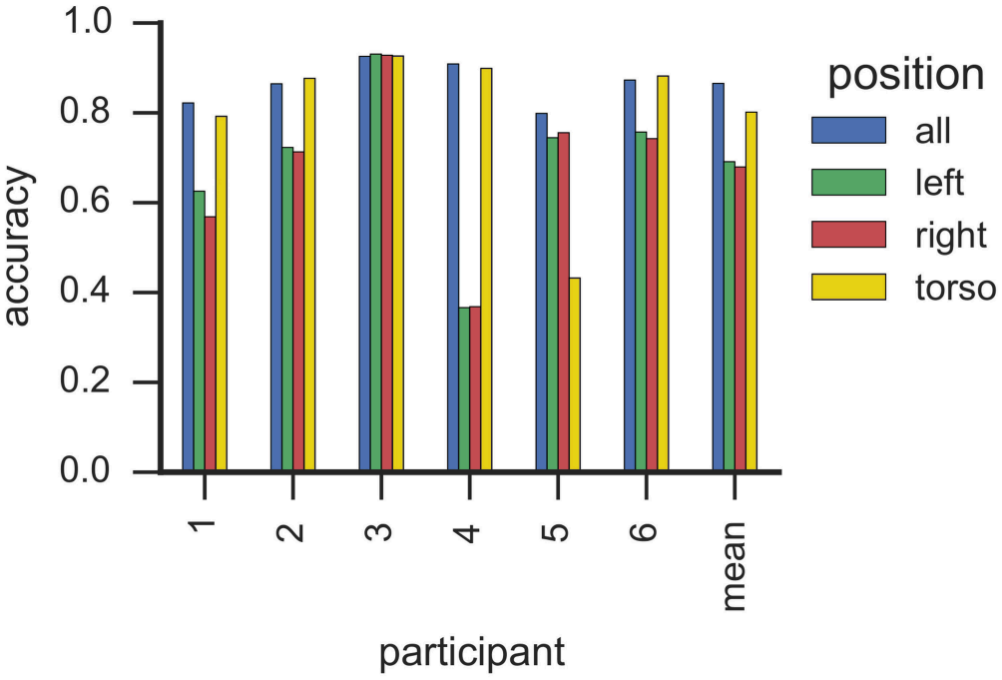
**This plot shows the classification accuracy based on sensor position with RF classifiers**. Each bar corresponds to one sensor from one participant. Sensors are grouped by participants, where the right-most group summarizes the average accuracy per sensor. We estimated the accuracy by means of *k*-fold cross-validation where folds correspond to recording sessions.

Figure 5 illustrates classification accuracy yielded for each individual sensor position as well as all sensor positions combined. On average, classifiers that used data only from the torso sensor reached comparable classification accuracy (>0.8) to a model trained with features from all sensors. In contrast, classifiers utilizing data from a single wrist sensor yielded lower classification accuracy on average (<0.7). However, these results differ substantially between participants. For example, data from participant 5 can be classified with high accuracy when all or only right-wrist sensor is used, while utilizing data from the torso sensor resulted in a decrease in accuracy. In contrast, a classifier trained for participant 2 with only data from the torso sensor yielded highest classification accuracy from all evaluated sensor configurations. This is particularly important since participant 2 showed 209 flap bouts and only one *rock* or *flaprock* bout (cf. Table 3), i.e., the SMM symptoms of participant 2 were almost exclusively hand movements rather than upper-body movements, and yet were better detected by a sensor worn on the torso. For participant 6, whose majority of SMMs are *rock* or *flaprock* events, data collected from the torso sensor only also yielded higher accuracies than data from a single wrist sensor or the combination of all available sensors. Participants 3 and 4 show a more evenly distributed weighting of SMM classes. Results from those participants also indicate that using exclusively data from the torso sensor reached higher classification accuracies than the wrist worn sensors.

Taken together, these findings suggest that reducing the sensor array to a single torso mounted sensor has little impact on recognition—even when detecting hand flapping. This is an important finding, suggesting that a single sensor could be used to recognize SMM, which is less burdensome and more likely to be accepted by participants with ASD who suffer from sensory sensitivities. One possible explanation for high classification accuracy using only a torso sensor relates to body mechanics. Due to the mechanical coupling of the body, arm motions may be propagated to the torso muscles, where they are picked up as movements of smaller amplitude by the torso accelerometer. Similar observations were noted in earlier studies (Min, 2014).

### 6.5. Feature importance

**Figure 6:**
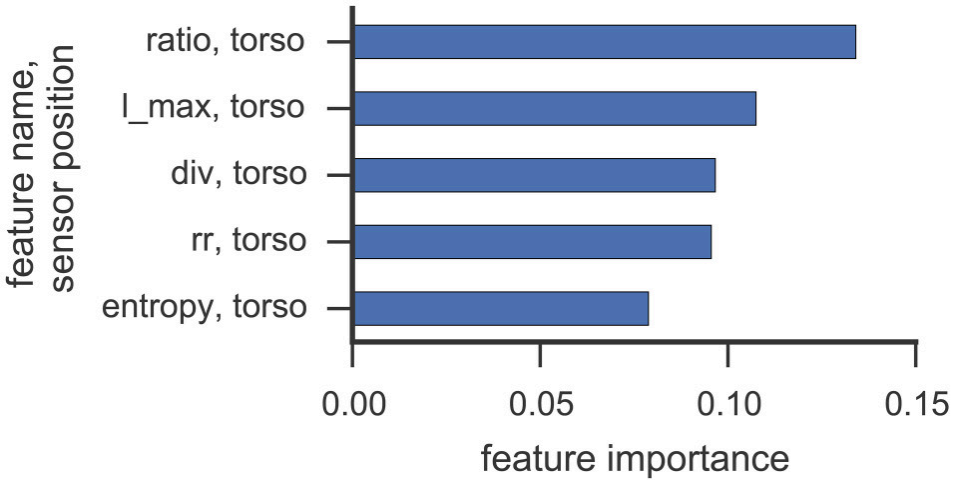
**This figure illustrates the five most important features yielded by the RF classifier for data collected from participant 2**. Each bar corresponds to a single feature. RQA features were extracted from a sensor position (right, left, torso) and the optimal ε value found in cross-validation was used.

**Figure 7:**
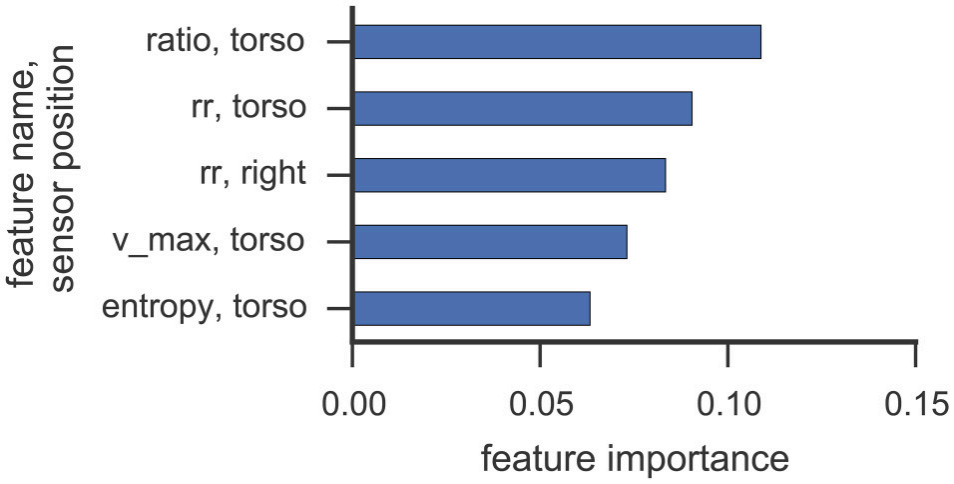
**This figure illustrates the five most important features yielded by the RF classifier for data collected from participant 4**. Each bar corresponds to a single feature. RQA features were extracted from a sensor position (right, left, torso) using the optimal ε value found in cross-validation.

Figures 6, 7 illustrate which features in the RF were ranked as the five most important for data collected from participant 2 and 4, respectively. For both participants, the 5 most important features were associated with the torso region.

The most important RQA features for participant 2 were *ratio* and *longest diagonal line* features, while for participant 4 *ratio* and *recurrence rate* lead the list of important features. Even though only one *rock* event was observed from participant 2 in Study 1 (cf. Table 3), features from the torso sensor are still ranked most important by the RF classifier. Participant 4 shows a more uniform class distribution between hand and upper-body related SMMs. Here, the feature importance measure of the RF classifier also indicates that features from the torso sensor contributed most to recognizing and classifying SMM events accurately. The *ratio* feature, as well as the *maximal diagonal length*, are both associated with the length distribution of vertical lines in the recurrence plots. The high ranking of *recurrence rate* features suggests that the number of recurrences in a recurrence plot is already a good indicator of a trajectory associated with SMM. This further emphasizes that the number and length of similar SMM segments in torso-based acceleration measurements is a reliable and valid indicator of SMM in individuals with ASD.

## 7. Conclusion

In this paper, we introduce a new set of features based on recurrence plot and recurrence quantification analysis that are able to capture the non-linear nature of SMM in individuals with ASD despite sensor orientation. By using the new feature set on an existing corpus of data that involved three 3-axis accelerometers, we achieved between 5 and 9% increase in accuracy compared to current state-of-the-art published results. The results also indicate that our approach allows us to recognize SMM in a leave-one-participant-out fashion. Furthermore, at least for some participants, a few tens of samples in the training set are sufficient to achieve high detection accuracy on data from participants left out from classifier training. We also identified that the most useful features for classification were obtained from the accelerometer mounted on the torso. This suggests the potential for using only a single torso sensor to detect both body rocking and hand flapping in a reliable and valid way. In contrast to the wrist sensors, accuracy achieved when only using the torso sensor was almost as high as when all sensors were used in classification. If replicated, these findings would suggest that simpler sensor deployments could be used while still achieving automated multi-class SMM recognition with high accuracy. This is an important discovery with the potential to increase end user acceptance and thereby better facilitating wider scale deployments of accelerometers in the ASD population to evaluate functional significance of SMM and their response to intervention. To overcome limitations of our current analysis, future research should incorporate a larger data set from a wider ASD population to address differences in age or gender.

## Ethics statement

The study that generated the data featured in the paper was reviewed and approved by IRB Committees at the University of Rhode Island and the Groden Center.

## Author contributions

Conceived and designed the experiments: MG. Designed analytical approach: UG, NM, VM, MG, GP. Analyzed the data: UG, NM, VM. Contributed analysis tools: AS, SN, AB. Wrote the paper: UG, NM, VM, GP, AB, MG. Contributed to the contents and structure of the paper: UG, NM, VM, MG, GP, AB, SN, AS. Proofread and corrected manuscript: UG, NM, VM, MG, GP, AB, SN, AS.

### Conflict of interest statement

The authors declare that the research was conducted in the absence of any commercial or financial relationships that could be construed as a potential conflict of interest.

## Footnotes

^1^Here it should be noted that Plötz et al. (2012) did not aim to detect SMM, but rather more general challenging behaviors such as aggression, tantrums, and property destruction seen in ASD.
